# Simple mucinous cyst: another potential cancer precursor in the pancreas? Case report with molecular characterization and systematic review of the literature

**DOI:** 10.1007/s00428-021-03029-1

**Published:** 2021-01-28

**Authors:** Anna Caterina Milanetto, Alice Sabrina Tonello, Giovanni Valotto, Giada Munari, Claudio Luchini, Matteo Fassan, Claudio Pasquali

**Affiliations:** 1 Clinica Chirurgica 1 - Pancreatic and Endocrine Digestive Surgical Unit, Department of Surgery, Oncology and Gastroenterology, University of Padua, via Giustiniani, 2, 35128 Padua, Italy; 2 Veneto Institute of Oncology, IRCCS, Padua, Italy; 3 Department of Diagnostics and Public Health, Section of Pathology, University of Verona, Verona, Italy; 4 Department of Medicine, University of Padua, Padua, Italy

**Keywords:** Pancreas, Pancreatic cancer, Pancreatic cyst, Cystadenoma mucinous

## Abstract

Cystic lesions of the pancreas may range from benign to precursors of pancreatic cancer. Simple mucinous cyst (SMC) is larger than 1 cm, has a gastric-type flat mucinous lining, and minimal atypia without ovarian-type stroma. We report a new case of pancreatic SMC, coupling a systematic review of the English literature mainly focused on their clinic-pathological features. We reviewed 103 cases of SMC in adults (73 women), averaging 57 (range, 26–70) years. The SMCs were located in the body-tail region of the pancreas in 60 (58%) cases, presenting as single cystic lesions in 94% of cases; 43% of patients were asymptomatic. A preoperative fine-needle aspiration of the cyst fluid detected amylase and carcinoembryonic antigen positivity in 71% and 76% of cases, respectively. Patients underwent surgery mostly for suspected malignancy; in 83% of cases, a standard pancreatic resection was performed. Mean SMC size was 4.9 (range, 1.5–12.0) cm. Mucins MUC5AC and MUC6 resulted positive in 77% and 81% of cases performed, respectively, whereas MUC2 was negative in all but one patient. The SMC from our institution was characterized by a *KRAS* somatic mutation. The diagnosis of SMC should be considered when a solitary pancreatic cyst larger than 1 cm is detected in asymptomatic patients. To establish a correct diagnosis, an extensive histologic/immunohistochemical analysis is essential. The presence of a *KRAS* mutation highlights that SMC may represent another potential pancreatic cancer precursor.

## Introduction

Cystic lesions of the pancreas comprise a wide spectrum of lesions, ranging from benign to pre-neoplastic entities. The most frequent lesions include intraductal papillary mucinous neoplasms (IPMNs), mucinous cystic neoplasms (MCNs), serous cysts, and solid pseudopapillary neoplasms [1]. Recently, IPMN and MCN have been definitively indicated as precursors of pancreatic ductal adenocarcinoma [2–4]. In the last decades, the increasing availability of cross-sectional imaging allowed the detection of cystic pancreatic lesions in asymptomatic patients, leading to challenging differential diagnoses, especially in adults. Among benign cystic lesions, the so-called true cysts of the pancreas are rare. They may be congenital or acquired, and either solitary or multiple lesions. Congenital pancreatic cysts may exist alone or in association with von Hippel-Lindau syndrome or polycystic kidney disease [5]. Most of the single true cysts of the pancreas have been found in children, in the first few years of life [5]. These lesions have been addressed with different terms in the literature. Recently, there has been a nomenclature change [6], and the term “simple mucinous cyst” (SMC) has been recommended to describe cysts larger than 1 cm having gastric-type flat mucinous lining and at most minimal atypia without ovarian-type stroma [6]. However, these recommendations have not been maintained in the 2019 WHO classification, where this category is still lacking. Here we report a new case of SMC of the pancreas arisen in an adult patient, also coupling a systematic review of the English literature mainly focused on their clinic-pathological features.

## Case report

**Fig. 1:**
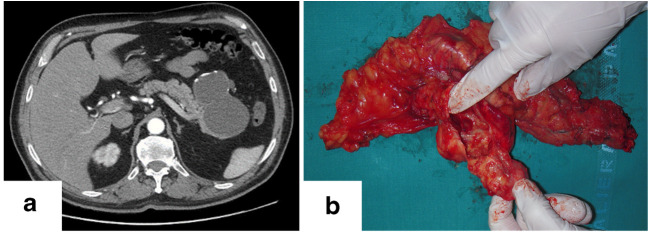
Computed tomography scan (**a**) and gross examination (**b**) showing a large unilocular cystic mass within the pancreatic body (diameter 8.7 cm)

A 63-year-old male presented with a 2-year history of abdominal pain without weight loss. There was no history of alcohol abuse, pancreatitis, or gallstone disease. An abdominal ultrasound showed a large cyst with internal septa and a thickened wall in the pancreatic area, over the left kidney. Magnetic resonance imaging (MRI) and computed tomography scan (Fig. 1a) confirmed the presence of a cystic lesion of 9.5 cm, located in the pancreatic tail. It appeared as a multilocular cyst with intraluminal septa and calcific spots within the irregular wall. The cystic lesion had no mural nodules or connection with the main pancreatic duct, which showed a regular diameter. The remaining pancreas had a normal appearance. At 18F-FDG positron emission tomography/computed tomography, the lesion showed no tracer uptake. Serum carcinoembryonic antigen (CEA) was high (30.7 ng/ml), whereas carbohydrate antigen 19.9 (CA19.9) was within the normal range. A mucinous cystic neoplasm was suspected, and the patient underwent distal pancreatectomy.

**Fig. 2:**
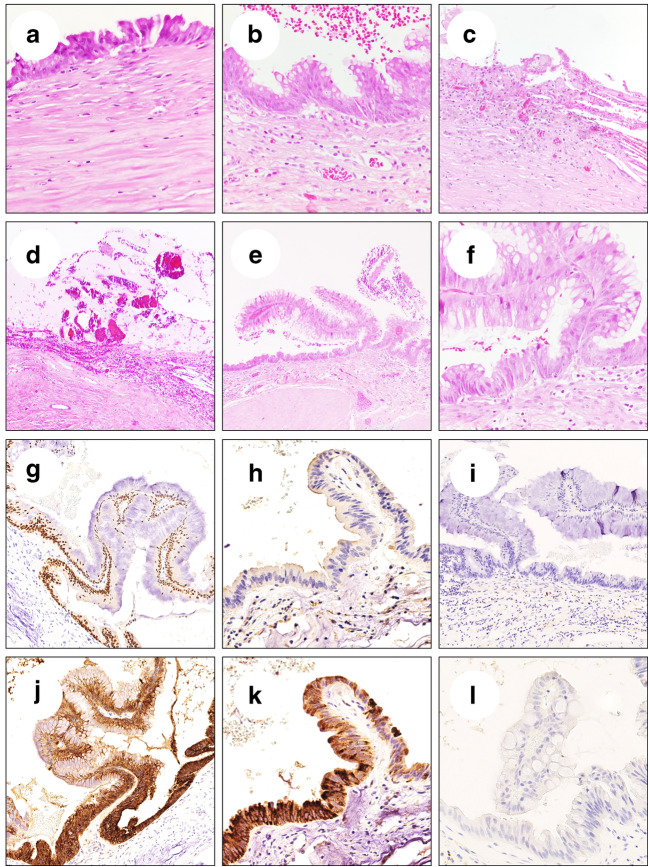
(**a**, **b**) Representative H&E pictures showing the flat mucin-producing gastric-type epithelium with focal epithelial folds; note the subepithelial fibrotic stroma without any evidence of ovarian-type stroma. The inner surface of the cyst was characterized by large areas of erosion/ulceration of the epithelium with mucin deposition (**c**, **d**); a single papillary projection was observed (**e**, **f**). Immunohistochemical analysis of the lesion showed positive staining for CDX2 (**g**), negative for CD10 (**h**) and MUC1 (**i**), positive for MUC4 (**j**) and MUC5AC (**k**), and negative for MUC6 (**l**). (Original magnifications ×10, ×20, and ×40)

Gross examination revealed a large unilocular cystic mass within the pancreatic body (diameter 8.7 cm) (Fig. 1b). The cyst did not present any communication with the main pancreatic duct, which was displaced by the mass. The lesion contained mucin material and was characterized by a thin fibrous wall, occasionally comprehending calcifications. The internal surface was smooth with some limited irregularities. The lesion was entirely sampled for histological analysis. At histology, the lesion was lined by a flat mucin-producing gastric-type columnar epithelium showing low-grade dysplasia, with focal epithelial folds and a single papillary projection. The surface was characterized by large areas of erosion/ulceration of the epithelium. No ovarian-type stroma was detected or peculiar mitotic activity (Fig. 2).

Immunohistichemical (IHC) analysis was automatically performed on 3–4-μm-FFPE sections using the Bond Polymer Refine Detection kit (Leica Biosystems, Newcastle Upon Tyne, UK) in the BOND-MAX system (Leica Biosystems). Staining was obtained using the following antibodies: EMA (clone E29; Biocare Medical, Pacheco, CA), CK7 (clone OV-TL 12/30; Cell Marque, Rocklin, CA), CK8-18 (clone 5D3; Leica Biosystems), CDX2 (clone epr2764y; Cell Marque), MUC4 (clone 8G7; Santa Crus Biotechnology, Dallas, TX), MUC5AC (clone CLH2; Leica Biosystems), CD10 (clone 56C6; Leica Biosystems), MUC 1 (clone Ma695; Leica Biosystems), MUC6 (clone CLH5; Leica Biosystems), chromogranin (clone DAK-A3; Agilent Biotechnologies, Santa Clara, CA), alpha-inhibin (clone R1; Agilent Biotechnologies), and progesterone receptor (clone NCL-PGR-312; Leica Biosystems). Immunohistochemistry was positive for epithelial membrane antigen, cytokeratin (CK) 7, CK8-18, CDX2, MUC4, and MUC5AC and negative for CD10, MUC1, MUC6, chromogranin, alfa-inhibin, and progesterone-receptor (PR).

**Fig. 3:**
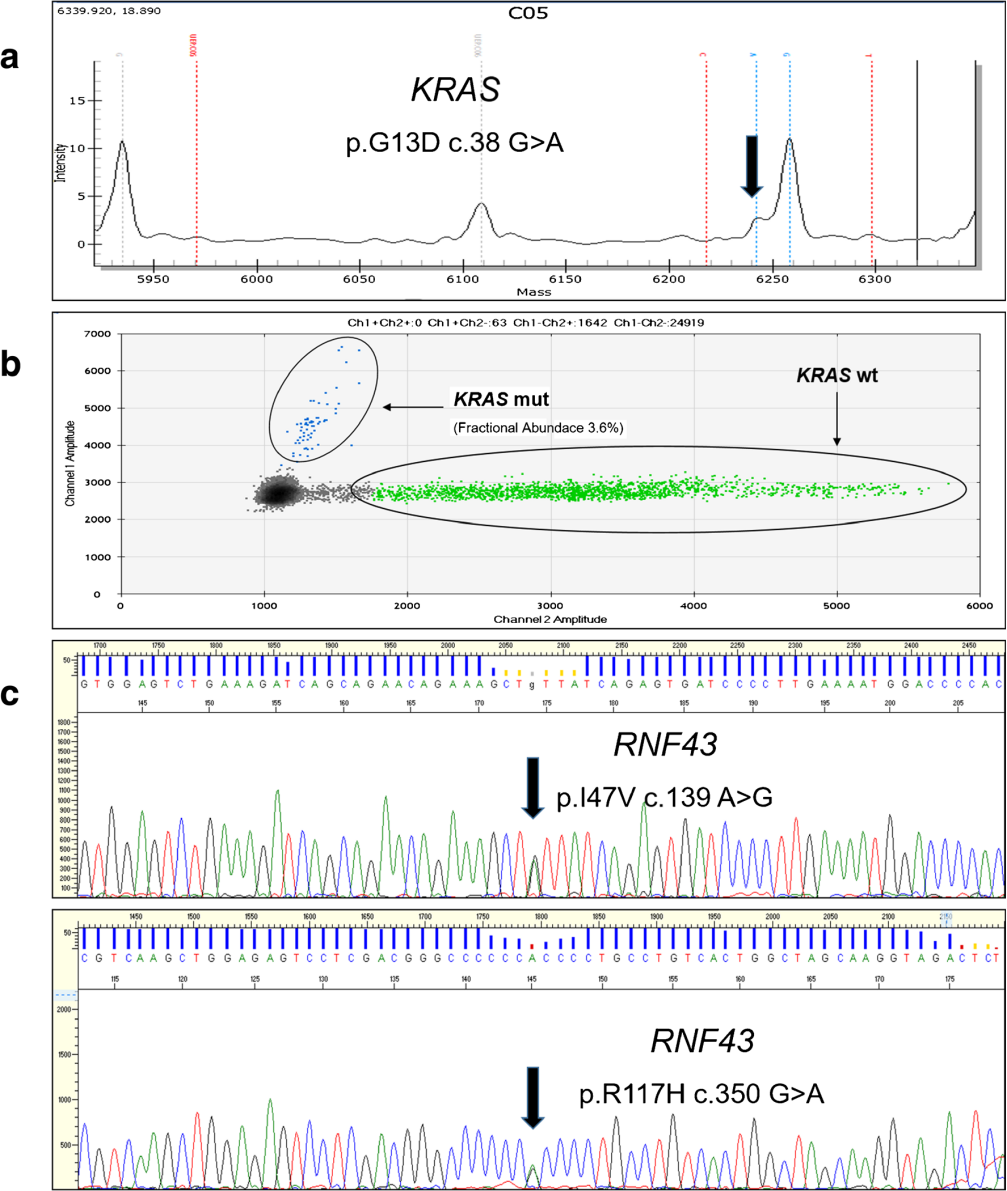
Representative Sequenom MassArray (**a**) and droplet digital PCR (**b**) output profiles of the *KRAS* gene mutation p.G13D; ddPCR was used to confirm the sequencing data due to the low prevalence of epithelial content in the microdissected material. (**c**) Representative Sanger chromatograms of the two germline single nucleotide variants (p.I47V and p.R117H) detected in the *RNF43* gene

We performed *RNF43*, *GNAS*, *RAS*, *BRAF*, and *PIK3CA* hotspot sequencing analysis after manual microdissection of the epithelial component of the cyst (Fig. 3). The lesion was characterized by a *KRAS* p.G13D somatic mutation, as detected by both Sequenom MassArray sequencing (Myriapod Colon Status, Diatech Pharmacogenetics, Jesi, Italy) and droplet digital PCR (Bio-Rad, Hercules, CA), and by two germline *RNF43* single nucleotide variants (i.e., p. I47V and p.R117H). The other evaluated genes did not present detectable alterations.

The most important differential diagnosis included MCN, a macrocystic/gastric-type IPMN, and a SMC of the pancreas. Of note, these lesions may show some overlapping features. The lack of an ovarian-type stroma, and in second line the fact that the patient was a male, helped in ruling out the diagnosis of MCN. The lack of a clear communication with the ductal tree, the absence of a diffuse papillary architecture, and its macroscopic appearance helped in excluding the diagnosis of IPMN. Therefore, a final diagnosis of SMC was achieved. The patient is still alive and without evidence of disease/relapse 42 months after surgical resection.

## Patients and methods

### Literature search

The PRISMA (Preferred Reporting Items for Systematic Reviews and Meta-analyses) guidelines were followed when performing and reporting this systematic review [7]. A systematic review was performed from January 1, 1984, to May 28, 2020, using a search string which included the terms “simple cyst,” “true cyst,” “solitary cyst,” “simple mucinous cyst,” and “mucinous non-neoplastic cyst” (Appendix).

### Inclusion criteria

Full-text studies published in English language after 1984 were included. In 1984, Cubilla et al. [8] firstly defined this congenital non-neoplastic cyst as a “simple cyst.” All publications related to pancreatic SMC (histologically confirmed), which included adult patients (defined as more than 18 years old) and reported a description of patient and tumor characteristics (demographics, diagnosis, treatment, and outcome), were considered for the eligibility phase.

### Studies selection and data extraction

Two investigators (A.S.T. and G.V.) independently reviewed all the records left after the screening phase. In case of disagreement, a consensus was reached involving a third investigator (A.C.M.). Case series including pediatric patients with aggregate clinical data were excluded. To avoid duplication of cases, the clinical data reported were cross-referenced by the country of origin, and then by the center from which the case originated. Variables that were recorded included patient age, gender, and symptoms; SMC location and size; pre-operative diagnosis (fine-needle aspiration-FNA or biopsy); type of surgery; histochemical and immunohistochemical data (CAM5.2, AE1/AE3, CK8-18, CK7, CK19, CK20, mucin-MUC1, MUC2, MUC5AC, MUC6, CEA, CA19.9, inhibin, PR); time of follow-up; and outcome.

## Results

### Literature selection and systematic review

The literature search generated 2121 reports, and after screening, 33 full-text articles met the inclusion criteria (Appendix). Three articles with English full-text not available were also excluded, and other 16 articles not reporting on immunohistochemical analysis were not eligible for the review. Finally, 14 studies [9–22] were included in the systematic review for qualitative synthesis. These included seven case reports and seven case series, and 12 out of 14 studies were published after 2000.

### Clinical findings

The case from our institution was included in the systematic review; thus, we reviewed 103 cases of SMC in adults (Table 1). There were 73 (71%) women and 27 men (not available gender, *n* = 3), averaging 57 (range, 26–70) years. Presenting symptoms were available in 68 (66%) cases. Twenty-nine (43%) patients were asymptomatic, whereas the other patients presented mainly with abdominal pain (27 cases). The SMCs were located in the body-tail region of the pancreas in 60 (58%) cases, and they presented as single cystic lesions in 97 (94%) cases. A preoperative FNA of the cyst fluid was performed in 70 patients, and biochemical analysis of cyst fluid and CA19.9 and CEA assessment were carried out. Amylase was positive in 17 out of 24 cases (71%) performed, whereas lipase resulted negative in all the 5 cases performed. In 58 cases, CEA was tested and resulted positive in 44 cases (76%), whereas CA19-9 was tested in only 6 cases, resulting positive in 5 cases. Finally, preoperative diagnosis was clearly stated only in 16 (16%) patients and consisted in a (mucinous) cystic neoplasm and/or a pseudocyst. The other patients underwent surgery mostly because of abdominal pain persistence after medical treatment, or because of a cystic pancreatic lesion increasing in size. Data on surgical treatment were available in 35 (34%) cases, and surgery consisted mainly in distal pancreatectomy (17 cases) and pancreatico-duodenectomy (11 cases). Parenchyma-sparing resections (enucleation, central pancreatectomy) were performed in six (17%) cases. Follow-up data were available for 59 (57%) patients, and all of them were alive and without evidence of disease after a mean follow-up time of 26 (range, 7–45) months.

**Table 1:** Clinic-pathological features, surgical treatment, and outcome (*n* = 103)

Author, year [ref]	Case of SMC (*n*)	Gender (*n*) Mean age ± SD (years)	Symptoms (*n*)	Pancreatic site (*n*)	Preoperative diagnosis (*n*)	Preoperative FNA (yes/no) Enzymes/tumor markers	Connection with the pancreatic duct system	Surgery (*n*)	Mean SMC size ± SD (cm)	HC and IHC stains positive	HC and IHC stains negative	Status Mean FU (range) (months)
Sperti et al., 1995 [9]	3	F (*n* = 2) M (*n* = 1) 60.3 ± 14.8	Abd pain (*n* = 3)	Body (*n* = 2) Tail (*n* = 1)	CN (*n* = 2) n.a. (*n* = 1)	Yes (*n* = 2) Amy−, Lip−, CEA−, CA19.9+	No (*n* = 3)	E (*n* = 2) DP (*n* = 1)	5.1 ± 3.9	CAM5.2, AE1/AE3	CEA, PAS, Alcian	Alive 23 (13–39)
Tanno et al., 1998 [10]	1	F 53	No	Tail	n.a.	Yes Amy−, Lip−, CEA−, CA19.9+	n.a.	DP	7	CA19.9	n.a.	Alive 45
Takahashi et al., 2001 [11]	1	F 50	No	Head	CN	Yes CA19.9+	No	PD	12	n.a.	PAS, Alcian	n.a.
Koshmal et al., 2002 [12]	5	F (*n* = 2) M (*n* = 3) 57.2 ± 18.9	No (*n* = 1), Abd pain (*n* = 2), Jaundice (*n* = 2)	Head (*n* = 3) Multiple (2)	n.a.	n.a.	No (*n* = 5)	n.a.	5.5 ± 3.3	CK7, CK8–18, CK19, CA19.9, MUC5AC (*n* = 4), CK20 (*n* = 4), MUC1 (*n* = 1)	MUC2, MUC6, CEA, inhibin	Alive 24 (12–96)
Brunner et al., 2004 [13]	1	M 58	Pancreatitis	Head	n.a.	Yes Amy+	n.a.	PD	3	MUC5AC, CK7, PAS, Alcian, CEA, CA19.9	MUC1, MUC2, CK20, inhibin	Alive 7
Fiamingo et al., 2005 [14]	1	F 26	Dyspepsia	Head	n.a.	Yes Amy−, Lip−	No	E	8	CA19.9	PAS	Alive 12
Cao et al., 2010 [15]	15	F (*n* = 12) M (*n* = 3) 59.6 (22–73)*	No (*n* = 7) Abd pain (*n* = 6) Polyuria (*n* = 1) Anorexia (*n* = 1)	Head (*n* = 7) Body (*n* = 5) Tail (*n* = 3)	n.a.	Yes CEA+ (*n* = 10), CEA− (*n* = 5)	No (*n* = 15)	PD (*n* = 7) DP (*n* = 5) CP (*n* = 2) TP (*n* = 1)	1.7 ± 0.9	PAS, MUC1 (*n* = 4), MUC5AC (*n* = 10)	MUC2	Alive (2–42)
Nadig et al., 2012 [16]	7	F (*n* = 6) M (*n* = 1) 70.3 ± 8.7	No (*n* = 4) Pancreatitis (*n* = 1) Abd pain (*n* = 2)	Head (*n* = 1) Tail (*n* = 2) Multiple (*n* = 4)	n.a.	Yes CEA+ (*n* = 5), CEA− (*n* = 2)	No	DP (*n* = 5) PD (*n* = 2)	1.8	MUC1 (*n* = 5), MUC5AC	MUC2	Alive 44 (up to 48)
Yang et al., 2013 [17]	1	F 69	No	Tail	n.a.	No	No	n.a.	3	CK7, CK19, MUC6, MUC1	MUC2, MUC5AC	n.a.
Zhu et al., 2013 [18]	23	F (*n* = 15) M (*n* = 8) 63.3 ± 7.6	n.a.	Head (*n* = 9) Body (*n* = 4) Tail (*n* = 10)	n.a.	Yes CEA+ (*n* = 14), CEA n.a. (*n* = 9), Amy+ (*n* = 16), Amy− (*n* = 1), Amy n.a. (*n* = 6)	No (*n* = 23)	n.a.	1.5 ± 0.1	CK7, CK20 (*n* = 1), PR (*n* = 2)	MUC2, inhibin	Alive (12–72)
Michalopoulos et al., 2014 [19]	1	F 47	Abd pain	Tail	CN	Yes Amy−, CEA−, CA19.9−	No	DP	6.6	CEA, CAM5.2	n.a	Alive 24
Krasinskas et al., 2017 [20]	39	F (*n* = 29) M (*n* = 7) n.a. (*n* = 3) 65 (22–85)*	No (*n* = 11) Abd pain (*n* = 12) Back pain (*n* = 3) Jaundice (*n* = 1) n.a. (*n* = 12)	Head (*n* = 11) Body/tail (*n* = 25) n.a. (*n* = 3)	Pseudocyst (*n* = 3) IPMN (*n* = 2) SCA (*n* = 1) n.a. (*n* = 33)	Yes (*n* = 17) CEA+ (*n* = 14)	Yes (*n* = 2) No (*n* = 22) n.a. (*n* = 15)	n.a.	2.4 (1.0–5.5)*	CK7 (*n* = 30) MUC5AC (*n* = 23) MUC6 (*n* = 28) MUC1 (*n* = 11) MUC2 (*n* = 1)	n.a	n.a.
Ishigami et al., 2017 [21]	3	F (*n* = 2) M (*n* = 1) 51.7 ± 9.4	No (*n* = 2) Jaundice (*n* = 1)	Tail (*n* = 1) Body (*n* = 2)	MCN	No	No (*n* = 3)	DP (*n* = 2) CP (*n* = 1)	4.7 ± 1.6	MUC1, MUC5AC	MUC2	n.a.
Kim et al., 2019 [22]	1	M 65	No	Body	MCN	Yes Amy−, Lip−, CEA+, CA19.9+	n.a.	DP	2.5	MUC1	MUC2	Alive 12
Present case	1	M 63	Abd pain	Tail	MCN	No	No	DP	8.7	EMA, CK7, CK8–18, CDX2, MUC4, MUC5AC	Inhibin, PR, CD10, MUC1, MUC6	Alive 42

*amy* amylase, *CA19.9* carbohydrate antigen 19.9, *CEA* carcinoembryonic antigen, *CK* cytokeratin, *CN* cystic neoplasm, *CP* central pancreatectomy, *DP* distal pancreatectomy, *EMA* epithelial membrane antigen, *E* enucleation, *F* female, *FNA* fine-needle aspiration, *FU* follow-up, *HC* histochemistry, *IHC* immunohistochemistry, *IPMN* intraductal papillary mucinous neoplasm, *lip* lipase, *M* male, *MCN* mucinous cystic neoplasm, *MUC* mucin, *n.a.* not available, *PAS* periodic acid–Schiff, *PD* pancreatico-duodenectomy, *PR* progesterone receptors, *SCA* serous cystic adenoma, *SMC* simple mucinous cyst, *TP* total pancreatectomy

*Range

### Pathological findings

Mean SMC size was 4.9 (range, 1.5–12.0) cm, reported either from imaging studies or from gross examination. No connection to the main or branch pancreatic ducts was demonstrated in 98% (83/85 reported) of cases. The immunohistochemical panels largely differed among the reported studies (Table 2). Cytokeratin7 was performed in 61 cases and resulted positive in 100% of cases. Other cytokeratins, such as CAM5.2, AE1/AE3, CK8-18, and CK19, resulted positive in all cases performed, even if they were tested in less than 10 cases. Among mucins, MUC5AC and MUC6 resulted positive in 77% and 81% of cases performed, respectively. Immunostaining for MUC1 was positive in 42% of cases, whereas MUC2 was negative in all but one patient (1% of cases). Other positive staining concerned CA19.9 which was positive in all 8 cases, whereas only 25% of cases resulted positive for CEA. The presence of ovarian-type stroma was excluded, with inhibin negative in all 30 cases performed, but a weakly positivity for PR was detected in two cases. Overall, no specific immunohistochemical marker has showed a significant diagnostic impact for this disease, and the diagnosis is still based on an accurate histological examination of the lesion.

**Table 2:** Histochemical and immunohistochemical stains (*n* = 103)

		Performed	Positive (%)
Alcian-PAS		21	16 (76)
Cytokeratins	CAM5.2	4	4 (100)
	AE1/AE3	3	3 (100)
	CK8–18	6	6 (100)
	CK7	61	61 (100)
	CK19	6	6 (100)
	CK20	29	5 (17)
Mucins	MUC1	62	26 (42)
	MUC2	86	1 (1)
	MUC5AC	62	48 (77)
	MUC6	36	29 (81)
Others	CEA	10	2 (25)
	CA19.9	8	8 (100)
	Alfa-inhibin	30	0 (0)
	PR	24	2 (8)

*CK* cytokeratin, *CA19.9* carbohydrate antigen 19.9, *CEA* carcinoembryonic antigen, *MUC* mucin, *PAS* periodic acid–Schiff, *PR* progesterone receptors

## Discussion

Cystic lesions of the pancreas with an epithelial wall without cell atypia were firstly described by Cubilla et al. [8] as “simple cysts” in the 1980s and slightly later as “true cysts” [5]. Later on, they have been defined as “mucinous non-neoplastic cysts” [12], due to the absence of cellular atypia. After the Baltimore Consensus Meeting 2014, the term “simple mucinous cyst” has been recommended to describe a cyst larger than 1 cm in size with predominantly flat (i.e., non-papillary) mucinous lining, with gastric phenotype, at most minimal atypia, and lacking ovarian-type stroma [6]. Although rare, SMCs may be congenital, and they may be associated with von Hippel Lindau syndrome or polycystic kidney disease. Most of the single pancreatic true cysts were found in children, in the first few years of life [5]. In adults, it is difficult to determine whether a cystic pancreatic lesion is a congenital benign cyst or not, especially in the absence of the abovementioned syndromes and when they are detected in asymptomatic patients in the fifth decade of life. In these cases, SMCs represent a challenging diagnosis, since a cystic neoplasm of the pancreas is firstly suspected.

The differential diagnosis of pancreatic SMCs comprises two main neoplasms with overlapping clinical and pathological features: IPMN and MCN (Table 3). The best imaging study for finalizing a differential diagnosis among pancreatic cysts is MRI. Typically, SMCs are unilocular or thinly septate at MRI, with internal signal intensity of simple fluid and no enhancing soft-tissue components [23]. In our review, SMCs presented as single lesions in 94% of cases; thus, it may be difficult to distinguish them from MCNs, especially when they appear as a large cyst with a thick wall [23]. Regarding MCNs (as defined by ovarian-type stroma), they arise almost always in female patients (>95%) and predominantly located in the body-tail region of the pancreas (>95%) [24]. Simple mucinous cysts have been previously reported to lack those two features [12], whereas other authors [15, 16] showed a SMC preponderance in women. In our review, SMCs emerged as a lesion typically located in the body-tail, but at lower prevalence than MCN (about 60% of cases), and affected women, again at lower prevalence than MCN (about 70% of cases). These features rendered the diagnostic characterization of SMCs even more difficult. When presenting as multiple cystic lesions, SMCs may be distinguished from IPMN for the lack of a connection with the pancreatic duct system. Finally, MRI can help in excluding other benign cystic lesions of the pancreas, such as a pseudocyst, which has typically a thick wall, and a retention cyst, which shows a connection with the duct system and a concomitant cause for ductal obstruction. Although endoscopic ultrasound may be a helpful tool in the diagnosis of cystic and solid pancreatic lesions, the analysis of the cyst fluid by endoscopic ultrasound-guided FNA is unable to distinguish SMCs from MCNs and IPMNs. In 70 patients, a FNA of the cyst fluid was performed, and amylase and CEA were positive in 71% and 76% of cases, respectively. As previously reported, measurement of CEA and amylase levels in the cyst fluid is not useful in distinguishing a SMC from IPMN and MCN [18], and CEA levels are effective in delineating a mucinous origin but are unable to discriminate malignant from benign lesions [25].

**Table 3:** Main clinic-pathological features characterizing pancreatic mucinous cystic lesion resembling simple mucinous cyst

Type of lesion	Demographic	Macroscopic	Microscopic	IHC	Molecular
SMC	No specific features in this category	No communications with the ductal tree; mainly unilocular	Mucinous epithelium, no papillary projection, no pseudo-ovarian stroma	MUC5AC+, MUC1 negative	*KRAS*
BD-IPMN	F = M, 5–7th decade	Communication with the ductal tree; multilocular	Papillary projections	MUC5AC +, MUC2+ if intestinal IPMN, MUC1+ if pancreatico-biliary IPMN	*GNAS*, *KRAS*, *ATM*, *RNF43*
MCN	F>>>M, 5–6th decade	No communications with the ductal tree; unilocular but also multilocular	Mucinous epithelium, pseudo-ovarian stroma	Epithelium: MUC5AC+, MUC1 negative; pseudo-ovarian stroma: SMA+, PR+, alfa-inhibin if luteinized cells	*KRAS*, *TP53*
Retention cyst	No specific features in this category	Unilocular	Ductal epithelium, may be focally mucinous, no papillary projection, no pseudo-ovarian stroma	It depends of the type of epithelium, in general MUC1+	No driver mutations

*BD-IPMN* branch duct intraductal papillary mucinous neoplasm, *MCN* mucinous cystic neoplasm, *SMC* simple mucinous cyst

In the present review, preoperative diagnosis was clearly stated only in 16% of patients, consisting mostly in a cystic neoplasm (i.e., IPMN, serous cystadenoma, and MCN) or a pseudocyst. Almost 70% of patients had symptoms (i.e., abdominal pain, jaundice), and the others had large or increasing in size cystic lesions. Therefore, surgery represented the appropriate treatment for symptoms relief and for suspected malignancy. Surgery consisted mainly in standard pancreatic resections (i.e., pancreatico-duodenectomy and distal pancreatectomy), whereas only 17% of patients underwent a limited pancreatic resection. Parenchyma-sparing techniques allow a good preservation of exocrine and endocrine pancreatic functions in the long term, and would be indicated in case of benign pancreatic lesions, irrespective of their size. In the present review, none of the patients had a correct preoperative diagnosis of SMC. Thus, the surgical choice was mostly restricted to standard resections, usually performed for oncological reasons, since a (mucinous) cystic neoplasm is usually considered at least pre-malignant [16].

At histology, SMCs usually appear as solitary and isolated unilocular cystic lesions lined by a single layer of cuboidal to columnar mucinous epithelium (without cytological atypia), supported by a hypocellular (not ovarian-type) stroma, not communicating with the pancreatic ductal tree [12]. In our review, immunohistochemical analysis showed positivity for CK7 in 100% of cases and also positivity for other cytokeratins (i.e., CK8-18, CK19). This finding is a common feature between SMCs and MCNs [12, 18], since both entities are lined by columnar mucin secreting cells [26]. In general, SMCs mainly have a gastric phenotype [27]. Among mucins, MUC1 immunostaining was positive in 42% of cases, similar to previously reported data [27]. Mucins MUC5AC and MUC6 resulted positive in 77% and 81% of cases, respectively; MUC5AC expression was previously described also in MCNs [12]. Simple mucinous cysts and MCNs may have a similar epithelial phenotype, but they differ in their stromal component and in their potential for malignant transformation [12]. In our review, the presence of ovarian-type stroma was excluded, since inhibin was negative in all 30 cases performed. However, two of these cases [18] showed a concomitant weak and focal positivity for PR. Although SMC stromal cells have been previously described as negative for PR [12], Zhu et al. [18] hypothesized that SMC may have a paucicellular fibrous stroma with focal and weak positivity for PR, which differs from the ovarian-type stroma diffusely and strongly positive for PR of MCNs [18]. On the other hand, it could be discussed whether the two cases with weak stromal positivity for PR might be two cases of MCNs characterized by the presence of atrophic stroma.

In our review, all but one case (1%) showed a negative MUC2 staining. Intestinal histological subtype IPMNs are often MUC2 positive (71–100%), in addition to being MUC5AC positive (100%) and mostly MUC1 negative; whereas gastric histological subtype IPMNs stain positive for MUC5AC but are negative for MUC2, like SMC [15, 27]. Therefore, since most BD-IPMNs are gastric histological subtype IPMNs, neither MUC5AC nor MUC2 are of use to distinguish BD-IPMN from SMC. As a result, distinguishing BD gastric-type low-grade IPMN from SMC may be very challenging, and only the lack of papillary projections can really support the diagnosis of SMC rather than IPMN in these cases. Moreover, the specific staining pattern of SMCs may be assessed only on surgically resected gross specimens; thus, at the moment, it is not useful in guiding the preoperative decision-making.

The pathogenesis and malignant potential of SMCs is still debatable. Krasinskas et al. [20] described that 55% of SMCs harbor *KRAS* mutations, and rare cases (8%) may harbor high-grade dysplasia. These findings support the hypothesis that SMCs may represent true neoplastic precursors [20]. On the other hand, a clonality assay indicated the polyclonal origin of the SMC epithelial cells, providing a strong experimental evidence of their non-neoplastic nature [15]. More recently, Attiyeh et al. [28] performed targeted sequencing analysis on 13 clinically and pathologically well-characterized SMCs and detected 59 mutations in 15 genes in the cohort, with a median of 4 mutations per cyst (range = 0–16 mutations per cyst). These findings underlined that the majority of SMCs can be considered in the spectrum of early, low-grade mucinous neoplasia. In the present review, none of the 59 patients with available follow-up data showed a disease recurrence, highlighting the low malignant potential of this tumor entity, although the median follow-up time (26 months) was relatively short.

In conclusion, the diagnosis of SMC should be considered when a solitary pancreatic cyst larger than 1 cm is detected in asymptomatic or paucisymptomatic patients. Simple mucinous cysts may overlap with MCNs and IPMNs in clinical, radiologic, and cyst fluid features. An accurate macroscopic sampling, coupled with an extensive histologic/immunohistochemical analysis, is essential for establishing the correct diagnosis. Lastly, the presence of a *KRAS* mutation highlights that SMC may represent another potential cancer precursor in the pancreas.

## Appendix. Search strategy

**PubMed: (Retrieved 2121 articles)**

(pancreas OR pancreatic) AND ((simple cyst) OR (true cyst) OR (solitary cyst) OR (simple mucinous cyst) OR (mucinous nonneoplastic cyst) OR “simple cyst” OR “true cyst” OR “solitary cyst” OR “simple mucinous cyst” OR “mucinous nonneoplastic cyst”)

*Filters applied:* English Language*. Custom date range: From 01/01/1984 to 05/28/2020.*
